# Associations of advanced liver fibrosis with heart failure with preserved ejection fraction in type 2 diabetic patients according to obesity and metabolic goal achievement status

**DOI:** 10.3389/fendo.2023.1183075

**Published:** 2023-10-24

**Authors:** Wangyan Jiang, Zhelong Liu, Shaohua Liu, Tingting Du

**Affiliations:** ^1^ Department of Endocrinology, Tongji Hospital, Tongji Medical College, Huazhong University of Science and Technology, Wuhan, Hubei, China; ^2^ Branch of National Clinical Research Center for Metabolic Diseases, Wuhan, Hubei, China; ^3^ Department of Clinical Nutrition, Deyang People’s Hospital, Deyang, Sichuan, China

**Keywords:** non-alcoholic fatty liver disease, advanced liver fibrosis, heart failure with preserved ejection fraction, metabolic goal achievement, type 2 diabetes mellitus

## Abstract

**Background:**

Heart failure with preserved ejection fraction (HFpEF), a major cause of morbidity and mortality in patients with type 2 diabetes mellitus (T2DM), is frequently coexisted with obesity, poor glycemic, blood pressure (BP), and/or lipid control. We aimed to investigate the associations of nonalcoholic fatty liver disease (NAFLD) and its advanced fibrosis with HFpEF according to obesity, glycated hemoglobin A1c (HbA1c), BP, and low-density lipoprotein cholesterol (LDL-C) goal achievement status in T2DM patients.

**Methods:**

A total of 2,418 T2DM patients who were hospitalized were cross-sectionally assessed. Liver fibrosis was evaluated by non-invasive biomarkers. Logistic regression analysis was used to evaluate the independent and combined associations of fibrosis status and diabetic care goal attainments with HFpEF risk.

**Results:**

Simple steatosis was not associated with HFpEF risk compared with patients without steatosis, while advanced liver fibrosis was found to have significantly higher odds for HFpEF risk (odds ratio,1.59; 95% confidence interval, 1.22-2.08). Advanced fibrosis in NAFLD was significantly associated with an increased risk of HFpEF, regardless of obesity status, HbA1c, BP, and LDL-C goal achievement status. P values for the interactions between fibrosis status and HbA1c control status, fibrosis status and BP control status, fibrosis status and LDL-C control status, and fibrosis status and body mass index (BMI) status on HFpEF risk were 0.021, 0.13, 0.001, and 0.23, respectively.

**Conclusion:**

In patients with T2DM, advanced hepatic fibrosis was significantly associated with HFpEF risk, irrespective of obesity status, HbA1c, BP, and LDL-C goal attainment status. Further, HbA1c and LDL-C goal attainment status modified this association.

## 1 Introduction

Heart failure (HF) is a major cause of morbidity and mortality in type 2 diabetes mellitus (T2DM) (1). T2DM patients initially progress to diastolic dysfunction (HF with preserved ejection fraction, HFpEF) and then to systolic dysfunction (HF with reduced ejection fraction, HFrEF) (2). Similar to HFrEF, HFpEF is associated with worse clinical prognosis and increased risk of hospitalization and mortality (3). HFpEF is more frequent in diabetic patients with obesity, poor glycemic, blood pressure (BP), and/or lipid control. Evidence showed that weight control and management of hyperglycemia, hypertension, and/or dyslipidemia are effective in preventing HF (4). Thus, the contribution of such modifiable risk factors to HFpEF risk is becoming a concern for its prevention.

Nonalcoholic fatty liver disease (NAFLD) has become one of the major liver diseases and a common indication for liver transplantation worldwide (5). The NAFLD epidemic has paralleled that of the diabetes epidemic. Approximately 60-70% of patients with T2DM suffered from NAFLD (6, 7). These two frequently overlapping diseases negatively influence each other’s prognoses. For example, T2DM increases the burden of NAFLD due to an increased risk of progression to non-alcoholic steatohepatitis (NASH), fibrosis, and cirrhosis (8). Vice versa, patients with NAFLD more commonly progress toward diabetic micro- and macro-vascular complications (9). Although one study has reported the associations of NAFLD and the severity of liver fibrosis with HFpEF in patients with T2DM (10), it included limited sample size. In addition, it remains unclear whether NAFLD and its advanced stages are associated with different risks of HFpEF in distinct populations defined by obesity status, glycated hemoglobin A1c (HbA1c), BP, and low-density lipoprotein cholesterol (LDL-C) goal attainment status.

In view of the links between diabetes care goal achievements and HFpEF (11, 12), and amid the ongoing NAFLD and diabetes epidemics, understanding the role of NAFLD and its advanced fibrosis as risk factors for HFpEF in different care goal attainment status is of clinical importance. Thus, we aimed to investigate how, independent of other risk factors, NAFLD and its advanced fibrosis and diabetic care goal achievements interactively influence HFpEF risk in T2DM patients.

## 2 Methods

### 2.1 Study design and population

**Figure 1:**
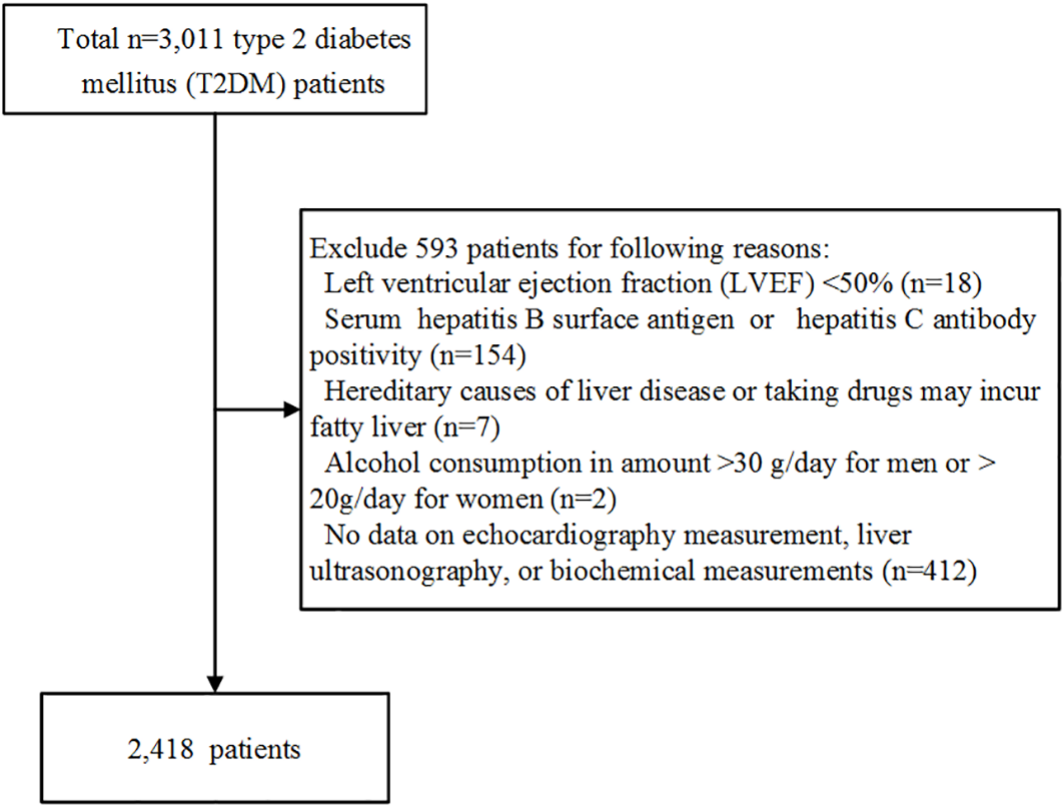
The flow diagram for the target population included in the study.

This cross-sectional study included 3,011 T2DM patients hospitalized in the Department of Endocrinology, Tongji Hospital, Tongji Medical College, Huazhong University of Science and Technology (Wuhan, China) between 2018 and 2021. T2DM was diagnosed according to the 2022 American Diabetes Association (ADA) criteria (13). Patients with implantable cardiac devices such as pacemakers, and implantable cardioverter-defibrillators were excluded to avoid HFpEF caused by tachycardiomyopahy. Patients with structural causes of HFpEF such as amyloidosis, hypertrophic obstructive cardiomyopathy, and dilated cardiomyopathy were also excluded. Further, we excluded 18 patients with left ventricular ejection fraction (LVEF) <50%, 154 patients with a positive hepatitis B surface antigen or hepatitis C antibody, 2 with excessive alcohol intake (>30 g/day for men and > 20g/day for women), and 412 with missing data on echocardiography measurement, liver ultrasonography, and/or biochemical measurements. Patients with hereditary causes of liver disease such as Wilson disease, and hereditary hemochromatosis, or taking drugs such as amiodarone, and corticosteroids that may incur fatty liver were also excluded. The remaining available 2,418 patients were included in the present analyses. The flow diagram for the target population in the study was shown in **Figure 1**.

According to the Private Information Protection Law, information that might identify patients was safeguarded by the Computer Center. This study was approved by the ethics committee of Tongji Hospital. Because we only retrospectively accessed a de-identified database for purposes of analysis, the informed consent requirement was exempted by the institutional review board. The procedures were followed in accordance with the ethical standards of the responsible committee on human experimentation and with the Helsinki Declaration of 1975, as revised in 1983.

### 2.2 Clinical measurements

Patients’ data including age, sex, height, weight, histories of current and previous illness, and medical treatments were obtained from medical records. Height, weight, waist circumference (WC), and BP were measured following standardized protocols from the World Health Organization (WHO). Patients’ seated BP was measured twice for every 5 min on the right arm after 5 min of rest with a sphygmomanometer. The mean of the two readings was used in data analysis. Body mass index (BMI) was calculated as weight (in kilograms) divided by the square of height (in meters).

Overnight fasting (for at least 8 h) blood samples were collected. All blood specimens were tested immediately after collection. HbA1c was measured using high performance liquid chromatography (D-10™; Bio-Rad Laboratories, Hercules, CA, USA). Fasting plasma glucose (FPG), triglycerides (TG), total cholesterol (TC), high-density lipoprotein cholesterol (HDL-C), LDL-C, alanine aminotransferase (ALT), aspartate aminotransferase (AST), and albumin (ALB) were measured on an autoanalyzer (Cobas C8000, Roche, Mannheim, Germany). Platelet (PLT) was assessed by sheath flow DC assay (XN-9000, Sysmex, Kobe, Japan). Hepatitis viral antigens/antibodies were detected with corresponding Architect reagents (Architect i2000, Abbott Diagnostics, Abbott Park, IL). All the blood measurements followed the same protocol.

### 2.3 Ultrasonography

Ultrasound tests were performed by certified sonographers using a high-resolution, real-time scanner (model SSD-2000; Aloka Co., Ltd., Tokyo Japan). All individuals were fasted overnight before imaging. Certified radiologists used standard criteria in evaluating the presence or absence of hepatic fat. Liver steatosis was defined based on the following criteria: the brightness of liver parenchyma, presence of liver to kidney contrast, presence of deep beam attenuation, and vascular blurring (14). Left ventricular end diastolic and end systolic volumes and ejection fraction (EF) were measured by 2-dimensional M-mode echocardiography at the apical two chamber and four-chamber views when patients were at rest. Transmitral peak early diastolic velocity (E) and peak late diastolic velocity (A) were measured by pulsed-wave Doppler echocardiography. Early annular diastolic tissue velocity (e’) was measured by pulsed-wave tissue Doppler echocardiography (15).

### 2.4 Definitions

According to the WHO Asia-Pacific guidelines (16), obesity was defined as BMI ≥ 25 kg/m^2^

According to the American Diabetes Association (ADA) criteria (17, 18), HbA1c goal achievement was defined as HbA1c level < 7.0%; BP goal achievement as systolic/diastolic BP < 130/80 mmHg; LDL-C goal achievement as LDL-C level < 100 mg/mL.

HFpEF was defined as EF ≥ 50% with either (1) E/A < 0.8, E/e’ < 8, and peak e’ < 10 cm/s, or (2) 0.8 < E/A < 1.5, 8 < E/e’ < 14, and peak e’ < 8 cm/s, or (3) E/A > 1.5, E/e’ >14 and peak e’ < 5 cm/s (19).

The presence of advanced liver fibrosis in patients with NAFLD was defined as the presence of a high probability for advanced fibrosis calculated by NAFLD fibrosis score (NFS) (20), BARD score (21), and fibrotic NASH index (FNI) (22), which have been extensively validated (23). The NFS was calculated as -1.675 + 0.037×age (years) + 0.094×BMI (kg/m^2^) + 1.13×impaired fasting glucose or diabetes (yes=1, no=0) + 0.99AST/ALT ratio-0.013×platelet count (10^9^/L) - 0.66×albumin (g/dL). Advanced fibrosis was defined as NFS ≥-1.455. The BARD score includes AST/ALT ratio ≥0.8 (2 points), BMI ≥28 kg/m^2^ (1 point), and the presence of diabetes (1 point). A score of ≥ 2 was associated with advanced fibrosis. The FNI was calculated as e^(-10.33+2.54*1nAST[*U/L*]+3.86*lnHbA1c[%]-1.66*lnHDL[*mg/dL*])^/1+e^(-10.33+2.54*1nAST[*U/L*]+3.86*lnHbA1c[%]-1.66*lnHDL[*mg/dL*])^ (22). Advanced fibrosis was defined as FNI >0.1. The homeostasis model assessment estimate of insulin resistance (HOMA-IR) was calculated using the following formula: HOMA-IR=fasting plasma glucose (mmol/L) ×fasting insulin (mIU/L)/22.5 (24).

### 2.5 Statistical analysis

All statistical analyses were conducted using the SPSS software (version 20.0 for Windows; SPSS, Chicago, IL, USA). Continuous variables were presented as means and standard deviations (SDs). Categorical variables were presented as percentages. One-way analysis of variance was applied to compare differences in means across groups. Bonferroni correction was applied to adjust P values for multiple comparisons. A Chi-square test was performed to assess differences of proportions across groups. Logistic regression models were used to estimate the associations (odds ratios [ORs], with 95% confidence intervals [CIs]) between the presence of NAFLD or advanced liver fibrosis and the risk of HFpEF. Three models were fitted. Model 1 was adjusted for age and sex. Model 2 was additionally adjusted for the duration of T2DM, antihypertensive drug use, lipid-lowering drug use, antidiabetic agent use, smoking, alcohol drinking, BMI, systolic BP, HbA1c, TG, and LDL-C. Model 3 was further adjusted for HOMA-IR. Further examination of the association of NAFLD and advanced liver fibrosis with HFpEF risk in BMI categories, HbA1c, BP, and LDL-C status were also evaluated. To assess if the associations differed by BMI status, logistic regression analysis was utilized to examine potential interaction effects between NAFLD/advanced liver fibrosis and BMI categories. The process was repeated separately for subgroups stratified by HbA1c, BP, and LDL-C status. Significance was accepted at a two-tailed P <0.05.

## 3 Results

Of the 2,418 T2DM patients included in the present analyses, the mean age was 52.9 years, and 62.8% were men. Nearly half of the T2DM patients (49.7%) suffered from NAFLD. The prevalence of NAFLD with advanced fibrosis defined by NFS and BARD was 29.5% and 35.2%, respectively. HFpEF was presented in 492 (20.3%) patients.

As shown in **Table 1**, T2DM patients with simple steatosis were younger, more likely to be male, and had higher BMI, WC, diastolic BP, AST, ALT, HbA1c, and adverse lipids than patients without NAFLD (all P value <0.05). T2DM patients with NFS-defined advanced fibrosis had even worse deteriorated anthropometric and cardiovascular risk profile. Further, they had the lowest septal e’ and E/A ratio, the largest IVSD and LVIDD, and were more likely to suffer from HFpEF (all P value <0.05). Similar findings were observed when BARD was used to define advanced fibrosis (**Supplementary Table 1**). HOMA-IR showed a positive relationship with NFS (r=0.017), BARD (r=0.018), and FNI (r=0.046).

**Table 1:** Characteristics of the study population according to NAFLD and advanced fibrosis status based on NFS.

	Total (n=2418)	Non-NAFLD n=1215 (50.3)	NAFLD without advanced fibrosis n=489 (20.2)	NAFLD with advanced fibrosis n=714 (29.5)
Age (years)	52.9 ± 12.9	55.5 ± 12.0	41.7 ± 11.9 ^*^	56.2 ± 10.6 ^†^
Male (%)	62.8	59.7	69.9 ^*^	63.2 ^†^
Smoking (%)	28.6	26.4	28.8	32.1 ^*^
Drinking (%)	19	17.9	16.4	22.8 ^*†^
Antihypertension drug (%)	36.1	36.5	20.9 ^*^	45.9 ^*†^
Lipid-lowering drugs (%)	12.7	13.1	8.4 ^*^	14.8 ^†^
Antidiabetic agents (%)	73.4	82.6	54.0 ^*^	70.9 ^*†^
Duration of diabetes ≥ 5 year (%)	50.6	59.9	28.2 ^*^	50.1 ^*†^
Systolic BP (mmHg)	131.7 ± 20.1	131.3 ± 21.2	130.5 ± 17.9	133.3 ± 19.5 ^†^
Diastolic BP (mmHg)	82.4 ± 12.4	80.3 ± 12.1	85.9 ± 12.6 ^*^	83.5 ± 11.8 ^*†^
BMI (kg/m^2^)	25.1 ± 3.7	23.7 ± 3.3	26.2 ± 3.7 ^*^	26.6 ± 3.4 ^*^
WC (cm)	92.9 ± 9.8	89.7 ± 9.2	94.9 ± 9.6 ^*^	96.9 ± 9.0 ^*†^
AST (IU/L)	22.6 ± 18.2	20.2 ± 14.2	25.9 ± 21.5 ^*^	24.5 ± 21.2 ^*^
ALT (IU/L)	27.2 ± 27.7	22.0 ± 25.1	39.2 ± 35.6 ^*^	27.7 ± 22.8 ^*†^
	Total (n=2418)	Non-NAFLD n=1215 (50.3)	NAFLD without advanced fibrosis n=489 (20.2)	NAFLD with advanced fibrosis n=714 (29.5)
PLT (10^9^/L)	224.4 ± 63.9	221.8 ± 64.8	273.8 ± 55.3 ^*^	194.9 ± 45.0 ^*†^
ALB (g/dL)	43.3 ± 4.2	42.5 ± 4.5	45.6 ± 3.4 ^*^	43.1 ± 3.5 ^*†^
TC (g/L)	4.6 ± 1.3	4.4 ± 1.2	5.0 ± 1.3 ^*^	4.6 ± 1.2 ^*†^
TG (g/L)	3.0 ± 2.9	2.2 ± 1.8	4.0 ± 3.9 ^*^	3.6 ± 3.2 ^*^
HDL-C (g/L)	1.0 ± 0.3	1.101 ± 0.3	0.958 ± 0.2 ^*^	0.960 ± 0.2 ^*^
LDL-C (g/L)	2.7 ± 1.0	2.7 ± 1.0	3.0 ± 1.0 ^*^	2.6 ± 0.9 ^†^
HbA1c (%)	9.3 ± 2.4	9.0 ± 2.5	9.9 ± 2.2 ^*^	9.4 ± 2.3 ^*†^
Echocardiographic information				
e’	7.9 ± 2.8	7.9 ± 2.9	8.8 ± 2.5 ^*^	7.4 ± 2.8 ^*†^
E/e’ ratio	10.5 ± 3.4	10.7 ± 3.6	9.5 ± 2.6 ^*^	10.8 ± 3.2 ^†^
E/A ratio	1.0 ± 0.4	0.96 ± 0.42	1.10 ± 0.34 ^*^	0.91 ± 0.36 ^†^
LAD (cm)	3.2 ± 0.4	3.20 ± 0.43	3.17 ± 0.36	3.29 ± 0.37^*†^
LVIDD (cm)	4.5 ± 0.4	4.47 ± 0.43	4.52 ± 0.37	4.53 ± 0.39*
IVSD (cm)	1.0 ± 0.4	0.99 ± 0.16	0.98 ± 0.12	1.01 ± 0.13^*†^
LVPWd (cm)	1.0 ± 0.2	0.98 ± 0.18	0.96 ± 0.13	0.99 ± 0.12^†^
EF (%)	66.1 ± 4.7	66.3 ± 4.8	65.7 ± 4.5 ^*^	65.9 ± 4.5
HFpEF (%)	20.3	18.1	19	25.1 ^*†^

Data are presented as means ± standard deviations.

Drinking was defined as light to moderate drinking (<15 and <30 g of alcohol per day for women and men, respectively).

NAFLD, nonalcoholic fatty liver disease; NFS, NAFLD Fibrosis Score; BP, blood pressure; BMI, body mass index; WC,

waist circumference; AST, aspartate aminotransferase; ALT, alanine aminotransferase; PLT, platelet count; ALB, albumin;

TC, total cholesterol; TG, triglycerides; HDL-C, high-density lipoprotein cholesterol; LDL-C, low-density lipoprotein cholesterol; HbA1c, hemoglobin A1c; e’, early diastolic velocity; E, early phase of mitral inflow; A, late phase of mitral inflow. LAD, left atrium diastole; LVIDD, left ventricular internal dimensions in diastole; IVSD, interventricular septal thickness at end diastole; LVPWd, left ventricular posterior wall thickness at end diastole; EF, ejection fraction; HFpEF, heart failure with preserved ejection fraction.

^*^p<0.05 compared with Non-NAFLD.

^†^p<0.05 compared with NAFLD without advanced fibrosis.

The ORs and 95% CIs for HFpEF from NAFLD and its advanced form compared with those without NAFLD were presented in **Table 2**. NAFLD was associated with an increased risk of HFpEF (model 1). Further adjustment for potential intermediate variables including BMI and HOMA-IR (model 3), NAFLD was still strongly associated with HFpEF risk (OR,1.50; 95% CI, 1.18 to 1.91; P=0.001). However, when NAFLD was stratified by NFS-defined advanced liver fibrosis, simple steatosis was no longer associated with HFpEF risk compared with patients without steatosis, while NFS-defined advanced liver fibrosis was found to have a significantly higher OR for HFpEF after sufficient adjustment (OR,1.59; 95% CI, 1.22 to 2.08; P=0.001) (model 3). When BARD or FNI was used to define advanced fibrosis, similar findings were observed (**Table 2**).

**Table 2:** Odds ratios (95% confidence intervals, CI) of the risk of HFpEF from NAFLD and its advanced fibrosis.

	Model 1		Model 2		Model 3	
	OR (95% CI)	P value	OR (95% CI)	P value	OR (95% CI)	P value
Non-NAFLD	1		1		1	
NAFLD	1.40 (1.14-1.72)	0.001	1.33 (1.06-1.67)	0.013	1.50 (1.18-1.91)	0.001
**NFS Score**						
Non-NAFLD	1		1		1	
NAFLD without advanced fibrosis	1.21 (0.90-1.63)	0.199	1.15 (0.84-1.57)	0.373	1.33 (0.95-1.86)	0.093
NAFLD with advanced fibrosis	1.50 (1.20-1.87)	0.000	1.43 (1.12-1.84)	0.005	1.59 (1.22-2.08)	0.001
**BARD Score**						
Non-NAFLD	1		1		1	
NAFLD without advanced fibrosis	1.37 (1.02-1.86)	0.039	1.32 (0.96-1.80)	0.084	1.35 (0.79-2.32)	0.273
NAFLD with advanced fibrosis	1.41 (1.13-1.76)	0.002	1.34 (1.05-1.72)	0.020	1.85 (1.18-2.90)	0.007
**FNI Score**						
Non-NAFLD	1		1		1	
NAFLD without advanced fibrosis	3.20 (0.53-19.5)	0.206	2.79 (0.42-18.3)	0.286	3.68 (0.47- 28.8)	0.214
NAFLD with advanced fibrosis	1.41 (1.15-1.73)	0.001	1.33 (1.06-1.67)	0.014	1.49 (1.17-1.91)	0.001

Model 1 was adjusted for age and sex.

Model 2 was further adjusted for duration of diabetes, antihypertensive drugs, lipid-lowering drugs, antidiabetic agents, smoking, alcohol drinking, body mass index, systolic blood, hemoglobin A1c, and serum levels of triglycerides and low-density lipoprotein based on model 1.

Model 3 was further adjusted for HOMA-IR based on model 2.

HFpEF, heart failure with preserved ejection fraction; NAFLD, nonalcoholic fatty liver disease; OR, odds ratio; CI, confidence interval. HOMA-IR, homeostasis model assessment estimate of insulin resistance; NFS, nonalcoholic fatty liver disease fibrosis score; FNI, fibrotic non-alcoholic steatohepatitis index.

Since comorbidities, such as chronic kidney disease (CKD) and cardio-cerebrovascular disease, that frequently coexisted with hospitalized diabetic patients were identified as important determinants that influence the short-term and long-term outcomes (25), we stratified our analysis by comorbidity status. The prevalence of CKD and cardio-cerebrovascular disease was 34.7%, and 13.6%, respectively. Advanced liver fibrosis was associated with an increased HFpEF risk, regardless of CKD status (**Supplementary Tables 2, 3**) and cardio-cerebrovascular disease status (**Supplementary Table 4, 5**).

We did a sensitivity analysis after excluding smokers. The results were essentially the same (**Supplementary Table 6**).

**Table 3** listed the independent and joint associations of NAFLD status and diabetic care goal attainments with HFpEF risk. After adjusting for potential intermediate variables including HOMA-IR, NFS-defined fibrosis remained a significant predictor of HFpEF, irrespective of HbA1c goal attainment status (OR, 2.65; 95% CI, 1.43 to 4.91; P <0.001 in subjects with fibrosis but optimal HbA1c control, and OR, 1.97; 95% CI, 1.27 to 3.06; P <0.001 in subjects with fibrosis and poor HbA1c control). The higher OR in patients with optimal HbA1c but with liver fibrosis than in patients with poor HbA1c control but without liver fibrosis (OR, 1.78; 95% CI, 1.09 to 2.90; P <0.001) and the significant interactions between fibrosis status and HbA1c control status on the risk of HFpEF (P=0.02) indicated that the association between liver fibrosis and HFpEF was modified by HbA1c goal attainment status.

**Table 3:** Odds ratios (95% confidence intervals) of the independent and joint associations of NAFLD and its advanced fibrosis based on NFS and diabetic care goal attainment status for HFpEF risk.

	Non-NAFLD	NAFLD without advanced fibrosis	NAFLD with advanced fibrosis	P interaction
**HbA1c**				0.0206
HbA1c < 7.0%	1.00	1.13 (0.40-3.22)	2.65 (1.43-4.91)	
HbA1c ≥ 7.0%	1.37 (0.90-2.09)	1.78 (1.09-2.90)	1.97 (1.27-3.06)	
**BP**				0.1264
BP < 130/80mmHg	1.00	1.93 (1.13-3.30)	1.63 (1.04-2.54)	
BP ≥ 130/80mmHg	1.11 (0.79-1.56)	1.22 (0.80-1.87)	1.76 (1.22-2.54)	
**LDL-C**				0.0099
LDL-C < 100mg/dL	1.00	2.05 (0.90-4.66)	1.85 (1.01-3.37)	
LDL-C ≥ 100mg/dL	1.33 (0.85-2.08)	1.71 (1.02-2.86)	2.05 (1.29-3.26)	
**LDL-C**				<0.001
LDL-C < 70mg/dL	1.00	1.63 (0.98-2.72)	1.57 (1.07-2.31)	
LDL-C ≥ 70mg/dL	1.35 (0.96-1.89)	1.67 (1.10-2.55)	2.22 (1.54-3.21)	
**BMI**				0.2338
BMI < 25 kg/m^2^	1.00	1.88 (1.24-2.86)	1.80 (1.23-2.62)	
BMI ≥ 25 kg/m^2^	1.16 (0.82-1.64)	1.07 (0.69-1.67)	1.68 (1.23-2.30)	
**BMI**				0.06
BMI < 30 kg/m^2^	1.00	1.40 (0.99-1.97)	1.63 (1.25-2.13)	
BMI ≥ 30 kg/m^2^	1.41 (0.62-3.20)	1.22 (0.56-2.68)	1.83 (1.08-3.09)	

All analyses were adjusted for age, sex, duration of T2DM, antihypertensive drug use, lipid-lowering drug use, antidiabetic agent use, smoking, alcohol drinking, BMI, systolic BP, HbA1c, TG, LDL-C and HOMA-IR, except that the variable was used for stratifying subgroups.

NAFLD, nonalcoholic fatty liver disease; NFS, NAFLD Fibrosis Score; HFpEF, heart failure with preserved ejection fraction; HbA1c, hemoglobin A1c; BP, blood pressure; LDL-C, low-density lipoprotein cholesterol; BMI, body mass index; T2DM, type 2 diabetes mellitus; HOMA-IR, homeostasis model assessment estimate of insulin resistance.

The relative risks were higher in patients with NAFLD with significant NFS-defined liver fibrosis compared with subjects with NAFLD but without liver fibrosis or those without NAFLD, regardless of BP goal attainment status (OR, 1.63; 95% CI, 1.04 to 2.54; P <0.001 in subjects with fibrosis but optimal BP control, and OR, 1.76; 95% CI, 1.22 to 2.54; P <0.001 in subjects with fibrosis and poor BP control) (**Table 3**). The interactions between fibrosis status and BP goal attainment status on HFpEF did not reach statistical significance (P=0.13), indicating that the association of fibrosis with HFpEF did not differ by BP goal attainment status.

The relative risks were higher in patients with NAFLD with significant NFS-defined liver fibrosis compared with subjects with NAFLD but without liver fibrosis or those without NAFLD, regardless of LDL-C goal attainment status (OR, 1.85; 95% CI, 1.01 to 3.37; P <0.001 in subjects with fibrosis but optimal LDL-C control, and OR, 2.05; 95% CI, 1.29 to 3.26; P <0.001 in subjects with fibrosis and poor LDL-C control) (**Table 3**). Simple steatosis also increased the risk of HFpEF in patients with poor LDL-C control (OR, 1.71; 95% CI, 1.02 to 2.86; P <0.001). The P value for NAFLD status × LDL-C goal attainment status interaction was 0.01 (**Table 3**), indicating that the association between liver fibrosis and HFpEF was modified by LDL-C goal attainment status.

A goal of LDL-C< 2.6 mmol/L (<100 mg/dL) for diabetic patients who were at high risk and a goal of LDL-C<1.8 mmol/L (<70 mg/dL) for diabetic patients who were at very high risk is recommended (26). Since most patients included in our study had at least one cardiovascular risk factors, we repeated the analysis when LDL-C goal achievement was defined as LDL-C<70 mg/dl. The results were essentially the same (**Table 3**).

NFS-defined fibrosis was associated with an increased risk of HFpEF, irrespective of BMI status (OR, 1.80; 95% CI, 1.23 to 2.62; P <0.001 in subjects with fibrosis but BMI< 25 kg/m^2^, and OR, 1.68; 95% CI, 1.23 to 2.30; P <0.001 in subjects with fibrosis and BMI ≥25 kg/m^2^) (**Table 3**). In addition, subjects with simple steatosis and BMI< 25 kg/m^2^ (OR, 1.88; 95% CI, 1.24 to 2.86; P <0.001) also had an increased HFpEF risk compared with those without NAFLD and BMI< 25 kg/m^2^. The interactions between fibrosis status and BMI status on HFpEF did not reach statistical significance (P=0.23), indicating that the associations of fibrosis with HFpEF did not differ by BMI status. When obesity was defined as BMI ≥ 30 kg/m^2^. The results were essentially the same (**Table 3**).

The independent and combined associations of NAFLD status and diabetic care goal attainments with HFpEF risk showed similar patternswhen BARD or FNI was used to define advanced fibrosis (**Supplementary Tables 7, 8**).

## 4 Discussion

This is, as far as we are aware, the first report to describe the associations of hepatic steatosis and/or the presence of advanced liver fibrosis with HFpEF in T2DM patients according to obesity status and metabolic goal achievement status. We found that in patients with T2DM, advanced fibrosis in NAFLD was significantly associated with an increased risk of HFpEF, regardless of obesity status, HbA1c, BP, and LDL-C goal attainment status. Further, in patients with poor LDL-C control, simple hepatic steatosis and advanced liver fibrosis were associated with an increased HFpEF risk.

The associations of HFpEF with NAFLD have been established in both the general population and T2DM patients (10, 27–30). However, most of these studies did not examine its relationship with liver fibrosis (28, 29), which is the major prognostic factor in NAFLD. The risk of cardiovascular disease as well as liver-related morbidity and mortality increased exponentially during the transition of NAFLD to advanced liver fibrosis and cirrhosis (31). Recently, Lee et al. reported that liver fibrosis, but not steatosis, was independently associated with HFpEF in non-cirrhotic subjects (32). For patients with T2DM, only one study investigated the relationship between NAFLD and its advanced fibrosis with HFpEF (10) and reported that HFpEF was independently associated with liver fibrosis, but not with steatosis. However, no information was available regarding the association of liver fibrosis with HFpEF according to obesity status and metabolic goal achievement status in T2DM patients. In the present study, we verified the significant association of liver fibrosis with increased HFpEF risk in T2DM patients. Since optimal control of HbA1c, BP, and LDL-C has been shown to have effects on liver histology (33, 34) as well as diabetic microvascular and macrovascular complications in T2DM patients (34), we investigated whether the associations of NAFLD and its advanced fibrosis with HFpEF differed by HbA1c, BP, and LDL-C goal attainment status. We found that advanced hepatic fibrosis, but not simple steatosis, was significantly associated with HFpEF risk, irrespective of HbA1c and BP goal attainment status. Further, HbA1c and LDL-C goal attainment status modified this association.

Studies showed that poor or suboptimal glycemic control may cause microvascular remodeling and cardiac fibrosis (35) and thus is correlated with an increased risk of heart failure (36). In the present study, we can assess the impact of liver fibrosis on HFpEF in T2DM aside from glycemic control by adjusting the effect of HbA1c level in logistic regression. We also assessed this association in a subgroup with optimal HbA1c control. The significant and independent associations of liver fibrosis with HFpEF in the entire study patients as well as in the subgroup who achieved the HbA1c goal attainment in our study highlight that liver fibrosis, independent of hyperglycemia, predisposes T2DM patients to an increased HFpEF risk. Interestingly, we also found that liver fibrosis was associated with a greater OR for HFpEF in those with optimal HbA1c control than in those with poor HbA1c control, and that the OR was higher in patients with optimal HbA1c but with liver fibrosis than in patients with poor HbA1c control but without liver fibrosis. Although the mechanisms for this are not known, it may be explained partly by the glycemic fluctuations. A recent study showed that patients with low HbA1c but high glycemic variability had a worse left ventricular diastolic function than patients with high HbA1c but low glycemic variability (37). Emerging evidence showed that patients with T2DM and NAFLD usually require more intensive anti-diabetic therapies to achieve an optimal glycemic control (38).

Dyslipidemia frequently coexisted with T2DM (39), in which overproduction of very low-density lipoproteins commonly occurs (40). Dyslipidemia induced lipotoxicity may eventually cause increased lipid accumulation in cardiomyocytes (41), which may increase the epicardial fat thickness, and impairment in mitochondrial function (42, 43), which may alter the myocardial energy metabolism. Evidence showed that increased epicardial fat volume and impaired myocardial glucose uptake are well-established risk factors for myocardial remodeling and diastolic dysfunction (44, 45). In the present study, we found that simple steatosis as well as liver fibrosis increased the risk of HFpEF in patients with poor LDL-C control. Reports showed that lipid-lowering drug use can reduce epicardial fat thickness (46). Hence, lipid control is important in preventing HFpEF in T2DM. We also noted an additive effect of liver fibrosis and poor LDL-C control on HFpEF. Hence, liver fibrosis further increased the HFpEF risk in patients with poor LDL-C control.

The non-obese NAFLD phenotype has sparked interest because of its high prevalence (47). Reports convinced that non-obese NAFLD subjects had severe liver histology and cardiovascular risk profiles that were identical or even worse than obese NAFLD subjects (48). Reports showed that non-obese NAFLD was more prevalent in diabetic patients compared with the general population (49), suggesting that non-obese NAFLD contributes to a large share of the disease burden of diabetes. In the present study, we found that fibrosis was associated with an increased risk of HFpEF in both non-obese and obese patients. Further, simple steatosis in non-obese patients was also associated with an increased HFpEF risk. This suggested that steatosis and liver fibrosis in T2DM patients, even if they were not obese, might be identified as an indicator of the presence of HFpEF. One possible explanation for these results may be due to a decreased capacity for storing fat in adipose tissue in non-obese NAFLD patients (50). Alterations in fatty acid trafficking lead to ectopic fat deposition in heart (51).

**Figure 2:**
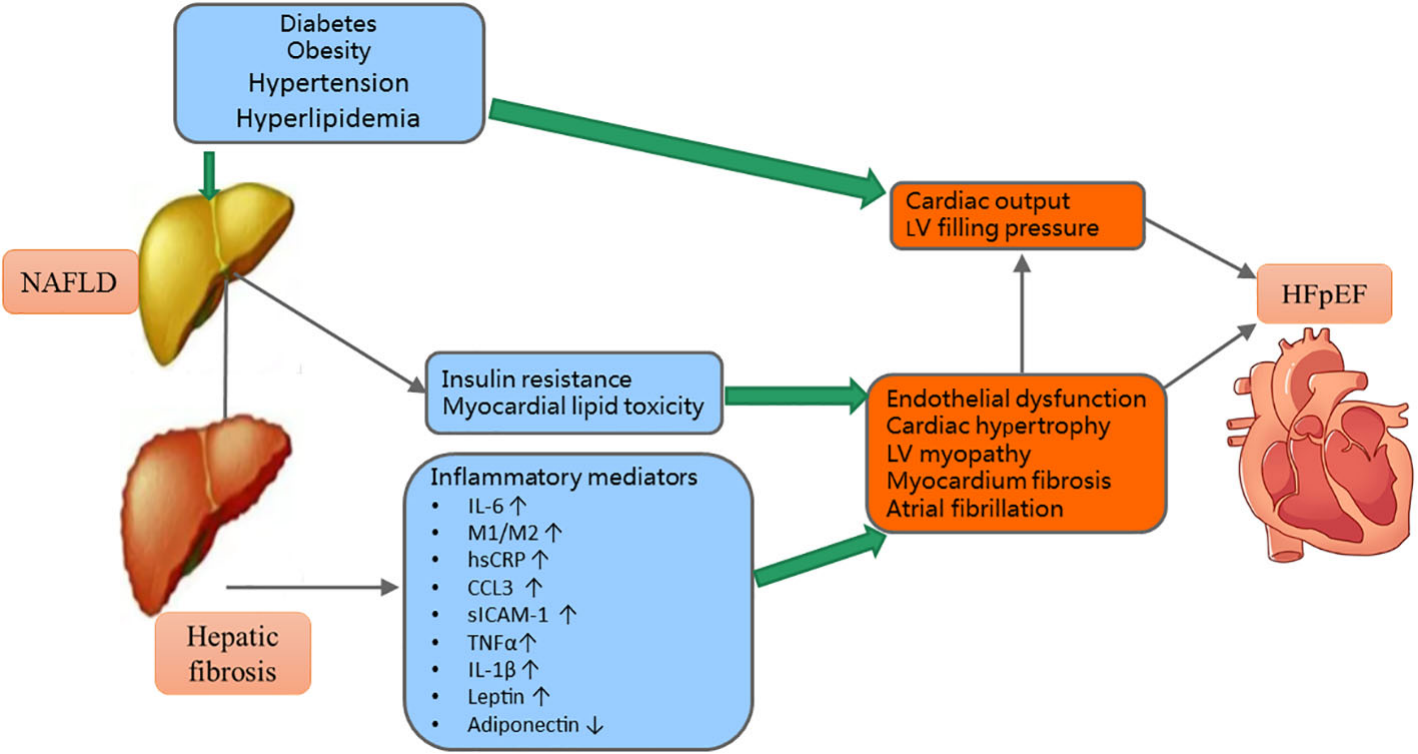
The mechanisms of the interactions of metabolic risk factors and hepatic fibrosis on HFpEF. NAFLD, nonalcoholic fatty liver disease; LV, left ventricular; HFpEF, heart failure with preserved ejection fraction; IL, interleukin; M1/M2, macrophage phenotype 1/2 ratio; hsCRP, high sensitivity C-reactive protein; CCL, chemokine ligand; sICAM, soluble intracellular adhesion molecule; TNF, tumor necrosis factor; ↑= increased; ↓= decrease.

The hypothetical pathogenesis of the interaction of fibrosis and metabolic risk factors on HFpEF was shown in **Figure 2**. Circulating inflammatory mediators are often increased in NAFLD patients (30, 52). For example, increased tumor necrosis factor (TNF)-α and interleukin (IL)-6 contribute to hepatocyte injuries. Then the damaged hepatocytes will release IL-33 that can promote heart profibrogenic (53, 54). Adipokines have a close relationship with metabolic risk factors and NAFLD. For example, leptin levels were significantly increased in NAFLD. The higher levels of circulating leptin were associated with increased severity of NAFLD (55). Further, the higher levels of circulating leptin can also incur cardiac hypertrophy and endothelial dysfunction (55).

The main strength of this study is the large number of T2DM patients included from an academic hospital. Further, we can get access to clinical, laboratory, and imaging data in medical records, which provided more in-depth clinical information that are not usually available in large epidemiological surveys.

There are several limitations. First, NAFLD was diagnosed by ultrasonography, which has a sub-optimal sensitivity in detecting mild-to-moderate steatosis, therefore missing patients with mild and/or moderate steatosis, rather than liver histopathology or other tools such as vibration-controlled transient elastography to quantify liver steatosis. Nevertheless, liver ultrasonography has been confirmed as an accurate and reliable tool for detecting fatty liver. Due to the relatively low cost and lack of radiation exposure, ultrasonography is widely used for identifying fatty liver in clinical settings and population studies. In the absence of liver biopsy to identify hepatic fibrosis, misclassification may have occurred. Second, although we adjusted for multiple potential confounding variables, residual and unmeasured confounding might not be fully addressed. Third, EF was measured by 2-dimensional M-mode echocardiography rather than the Simpsons method or the global longitudinal strain, which were more accurate in evaluating EF (56). Data on Tricuspid regurgitation velocity and left atrial volume index, which reflect left ventricular filling pressure, as well as data on septal, lateral, and atrial strain, which can reflect septal and atrial systolic and diastolic function (57, 58) were not available. Fourth, dynamic BMI, HbA1c, BP, and LDL-C changes during the process of diabetes management were not available, we cannot assess how these parameters change affect HFpEF risk. Finally, the cross-sectional study design makes it difficult to infer causality between the lipid parameters and NAFLD risk.

## 5 Conclusion

In conclusion, in patients with T2DM, advanced hepatic fibrosis was significantly associated with HFpEF risk, irrespective of BMI categories, HbA1c, BP, and LDL-C goal attainment status. Hepatic fibrosis increased the risk of HFpEF even in diabetic patients without obesity or achieving good HbA1c, BP, and LDL-C control.

## Data availability statement

The raw data supporting the conclusions of this article will be made available by the authors, without undue reservation.

## Ethics statement

The studies involving humans were approved by the ethics committee of Tongji Hospital. The studies were conducted in accordance with the local legislation and institutional requirements. Written informed consent for participation was not required from the participants or the participants’ legal guardians/next of kin in accordance with the national legislation and institutional requirements.

## Author contributions

WJ: data collection, statistical analyses, interpreting data, and writing of the draft manuscript; ZL: study design, interpreting data, and critical revision of the manuscript. SL: data collection; TD: study design, interpreting data, and critical revision of the manuscript. All authors contributed to the article and approved the submitted version.

## Glossary

**Table:**

NAFLD	Non-alcoholic fatty liver disease
HFpEF	Heart failure with preserved ejection fraction
T2DM	Type 2 diabetes mellitus
HbA1c	Glycated hemoglobin
BP	blood pressure
LDL-C	Low-density lipoprotein cholesterol
BMI	Body mass index
HF	Heart failure
ADA	American Diabetes Association
LVEF	Left ventricular ejection fraction
WC	Waist circumference
WHO	World Health Organization
FPG	Fasting plasma glucose
TG	Triglycerides
TC	Total cholesterol
HDL-C	High-density lipoprotein cholesterol
ALT	Alanine aminotransferase
AST	aspartate aminotransferase
ALB	Albumin
PLT	Platelet
EF	Ejection fraction
NFS	Non-alcoholic fatty liver disease Fibrosis Score
HOMA-IR	Homeostasis model assessment of insulin resistance
SDs	Standard deviations
OR	Odds ratios
CIs	Confidence intervals
NASH	Non-alcoholic steatohepatitis
FNI	Fibrotic NASH index
CKD	chronic kidney disease
IL	interleukin
M1/M2	macrophage phenotype 1/2 ratio
hsCRP	high sensitivity C-reactive protein
CCL	chemokine ligand
sICAM	soluble intracellular adhesion molecule
TNF	tumor necrosis factor
