# Supplementary material for: Associations of advanced liver fibrosis with heart failure with preserved ejection fraction in type 2 diabetic patients according to obesity and metabolic goal achievement status
Source: Front Endocrinol (Lausanne). 2023 Oct 24;14:1183075. doi: 10.3389/fendo.2023.1183075 (PMC10628500; doi:10.3389/fendo.2023.1183075)
Supplement: Supplementary file 1 [file Table_1.docx]

***Supplementary Material***

Supplementary table 1. Characteristics of the study population according to NAFLD and advanced fibrosis status based on BARD score.

|  | Non-NAFLD  n=1215 (50.3) | NAFLD without advanced fibrosis  n=351 (14.5) | NAFLD with advanced fibrosis  n=852 (35.2) |
| --- | --- | --- | --- |
| Age (years) | 55.5±12.0 | 46.9±12.1 ^*^ | 51.7±13.4 ^*†^ |
| Male (%) | 59.7 | 78.1 ^*^ | 60.9 ^†^ |
| Smoking (%) | 26.4 | 35.9 ^*^ | 28.6 ^†^ |
| Drinking (%) | 17.9 | 20.5 | 20.1 |
| Antihypertension drug (%) | 36.5 | 25.1 ^*^ | 40.1 ^†^ |
| Lipid-lowering drugs (%) | 13.1 | 10.3 | 13.0 |
| Antidiabetic agents (%) | 82.6 | 59.0 ^*^ | 66.1 ^*†^ |
| Duration of diabetes ≥ 5 year (%) | 59.9 | 37 ^*^ | 43 ^*^ |
| Systolic BP (mmHg) | 131.3±21.2 | 130.3±17.0 | 132.9±19.6 |
| Diastolic BP (mmHg) | 80.3±12.1 | 85.6±11.4 ^*^ | 84.0±12.5 ^*^ |
| BMI (kg/m^2^) | 23.7±3.3 | 25.0±1.8 ^*^ | 27.0±3.9 ^*†^ |
| WC (cm) | 89.7±9.2 | 93.5±6.3 ^*^ | 97.2±10.1 ^*†^ |
| AST (IU/L) | 20.2±14.2 | 25.2±15.7 ^*^ | 25.0±23.2 ^*^ |
| ALT (IU/L) | 22.0±25.1 | 44.0±31.3 ^*^ | 28.4±27.4 ^*†^ |
|  | Non-NAFLD  n=1215 (50.2) | NAFLD without advanced fibrosis  n=351 (14.5) | NAFLD with advanced fibrosis  n=852 (35.2) |
| PLT (10^9^/L) | 221.8±64.8 | 228.4±63.2 | 226.4±62.7 |
| ALB (g/dL) | 42.5±4.5 | 45.2±3.3 ^*^ | 43.7±3.7 ^*†^ |
| TC (g/L) | 4.4±1.2 | 4.8±1.2 ^*^ | 4.7±1.3 ^*^ |
| TG (g/L) | 2.2±1.8 | 4.0±3.4 ^*^ | 3.7±3.5 ^*^ |
| HDL-C (g/L) | 1.101±0.3 | 0.962±0.2 ^*^ | 0.959±0.2 ^*^ |
| LDL-C (g/L) | 2.7±1.0 | 2.8±1.0 | 2.8±1.0 |
| HbA1c (%) | 9.0±2.5 | 9.7±2.3 ^*^ | 9.6±2.3 ^*^ |
| Echocardiographic information |  |  |  |
| e’ | 7.9±2.9 | 8.4±2.4 ^*^ | 7.8±2.9 ^†^ |
| E/e’ ratio | 10.7±3.6 | 9.7±2.9 ^*^ | 10.5±3.1 ^†^ |
| E/A ratio | 0.96±0.42 | 1.04±0.33 ^*^ | 0.97±0.37 ^†^ |
| LAD (cm) | 3.20 ± 0.43 | 3.20 ± 0.34 | 3.26 ± 0.36 ^*†^ |
| LVIDD (cm) | 4.5 ± 0.4 | 4.48 ± 0.37 | 4.55 ± 0.38 ^*†^ |
| IVSd (cm) | 1.0 ± 0.4 | 0.99 ± 0.11 | 1.00 ± 0.14 |
| LVPWd (cm) | 1.0 ± 0.2 | 0.96 ± 0.09 | 0.99 ± 0.14^†^ |
| EF (%) | 66.3±4.8 | 65.9±4.6 | 65.8±4.5 ^*^ |
| HFpEF, n (%) | 18.1 | 21.9 | 22.9 ^*^ |

Data are presented as means and standard deviations.

Drinking was defined as light to moderate drinking (<15 and <30 g of alcohol per day for women and men, respectively).

NAFLD, nonalcoholic fatty liver disease; BP, blood pressure; BMI, body mass index;

WC, waist circumference; AST, aspartate aminotransferase; ALT, alanine aminotransferase; PLT, platelet count;

ALB, albumin; TC, total cholesterol; TG, triglycerides; HDL-C, high-density lipoprotein cholesterol; LDL-C,

low-density lipoprotein cholesterol; HbA1c, glycated hemoglobin; E, early phase of mitral inflow; e’, early diastolic

velocity; A, late phase of mitral inflow; LAD, left atrium diastole; LVIDD, left ventricular internal dimensions

in diastole; IVSD, interventricular septal thickness at end diastole; LVPWd, left ventricular posterior wall

thickness at end diastole; EF, ejection fraction; HFpEF, heart failure with preserved ejection fraction.

^*^p<0.05 compared with Non-NAFLD.

^†^p<0.05 compared with NAFLD without advanced fibrosis.

Supplementary table 2. Odds ratios (95% confidence intervals, CI) of the risk of HFpEF from NAFLD and its advanced fibrosis in patients without chronic kidney disease

|  | Model 1 |  | Model 2 |  | Model 3 |  |
| --- | --- | --- | --- | --- | --- | --- |
|  | OR (95% CI) | P value | OR (95% CI) | P value | OR (95% CI) | P value |
| Non-NAFLD | 1 |  | 1 |  | 1 |  |
| NAFLD | 1.57 (1.22-2.02) | 0.000 | 1.37 (1.04-1.81) | 0.027 | 1.61 (1.19 -2.18) | 0.002 |
| **NFS Score** |  |  |  |  |  |  |
| Non-NAFLD | 1 |  | 1 |  | 1 |  |
| NAFLD without advanced fibrosis | 1.40 (0.98-2.00) | 0.068 | 1.23 (0.85-1.80) | 0.276 | 1.45 (0.97-2.18) | 0.072 |
| NAFLD with advanced fibrosis | 1.66 (1.26-2.19) | 0.000 | 1.45 (1.06-1.97) | 0.019 | 1.70 (1.22-2.36) | 0.002 |
| **BARD Score** |  |  |  |  |  |  |
| Non-NAFLD | 1 |  | 1 |  | 1 |  |
| NAFLD without advanced fibrosis | 1.44 (1.00-2.06) | 0.050 | 1.33 (0.92-1.94) | 0.131 | 1.39 (0.93-2.09) | 0.112 |
| NAFLD with advanced fibrosis | 1.63 (1.24-2.14) | 0.000 | 1.39 (1.02-1.89) | 0.036 | 1.73 (1.25-2.41) | 0.001 |
| **FNI Score** |  |  |  |  |  |  |
| Non-NAFLD | 1 |  | 1 |  | 1 |  |
| NAFLD without advanced fibrosis | 1.79 (0.18-17.5) | 0.617 | 1.76 (0.17-18.3) | 0.636 | 2.26 (0.18-28.0) | 0.525 |
| NAFLD with advanced fibrosis | 1.57 (1.22-2.02) | 0.001 | 1.37 (1.04-1.81) | 0.028 | 1.61 (1.19-2.17) | 0.002 |

Model 1 was adjusted for age and sex.

Model 2 was further adjusted for duration of diabetes, antihypertensive drugs, lipid-lowering drugs, antidiabetic agents, smoking, alcohol drinking, body mass index, systolic blood, hemoglobin A1c, and serum levels of triglycerides and low-density lipoprotein based on model 1.

Model 3 was further adjusted for HOMA-IR based on model 2.

HFpEF, heart failure with preserved ejection fraction; NAFLD, nonalcoholic fatty liver disease; OR, odds ratio; CI, confidence interval. HOMA-IR, homeostasis model assessment estimate of insulin resistance; NFS, nonalcoholic fatty liver disease fibrosis score; FNI, fibrotic non-alcoholic steatohepatitis index.

Supplementary table 3. Odds ratios (95% confidence intervals, CI) of the risk of HFpEF from NAFLD and its advanced fibrosis in patients with chronic kidney disease

|  | Model 1 |  | Model 2 |  | Model 3 |  |
| --- | --- | --- | --- | --- | --- | --- |
|  | OR (95% CI) | P value | OR (95% CI) | P value | OR (95% CI) | P value |
| Non-NAFLD | 1 |  | 1 |  | 1 |  |
| NAFLD | 1.07 (0.75-1.52) | 0.719 | 1.22 (0.81-1.82) | 0.341 | 1.27 (0.82-1.96) | 0.279 |
| **NFS Score** |  |  |  |  |  |  |
| Non-NAFLD | 1 |  | 1 |  | 1 |  |
| NAFLD without advanced fibrosis | 0.89 (0.52-1.53) | 0.672 | 0.91 (0.51-1.62) | 0.730 | 1.01 (0.54-1.90) | 0.971 |
| NAFLD with advanced fibrosis | 1.15 (0.78-1.69) | 0.480 | 1.39 (0.90-2.15) | 0.142 | 1.39 (0.87-2.23) | 0.164 |
| **BARD Score** |  |  |  |  |  |  |
| Non-NAFLD | 1 |  | 1 |  | 1 |  |
| NAFLD without advanced fibrosis | 1.24 (0.70-2.18) | 0.460 | 1.25 (0.69-2.29) | 0.460 | 1.31 (0.70-2.47) | 0.403 |
| NAFLD with advanced fibrosis | 1.02 (0.70-1.49) | 0.904 | 1.20 (0.78-1.85) | 0.404 | 1.26 (0.79-2.01) | 0.342 |
| **FNI Score** |  |  |  |  |  |  |
| Non-NAFLD | 1 |  | 1 |  | 1 |  |
| NAFLD without advanced fibrosis | - | - | - | - | - | - |
| NAFLD with advanced fibrosis | 1.09 (0.76-1.56) | 0.643 | 1.19 (0.79-1.78) | 0.404 | 1.24 (0.80-1.91) | 0.340 |

Model 1 was adjusted for age and sex.

Model 2 was further adjusted for duration of diabetes, antihypertensive drugs, lipid-lowering drugs, antidiabetic agents, smoking, alcohol drinking, body mass index, systolic blood, hemoglobin A1c, and serum levels of triglycerides and low-density lipoprotein based on model 1.

Model 3 was further adjusted for HOMA-IR based on model 2.

HFpEF, heart failure with preserved ejection fraction; NAFLD, nonalcoholic fatty liver disease; OR, odds ratio; CI, confidence interval. HOMA-IR, homeostasis model assessment estimate of insulin resistance; NFS, nonalcoholic fatty liver disease fibrosis score; FNI, fibrotic non-alcoholic steatohepatitis index.

Supplementary table 4. Odds ratios (95% confidence intervals, CI) of the risk of HFpEF from NAFLD and its advanced fibrosis in patients without cardio-cerebrovascular disease

|  | Model 1 |  | Model 2 |  | Model 3 |  |
| --- | --- | --- | --- | --- | --- | --- |
|  | OR (95% CI) | P value | OR (95% CI) | P value | OR (95% CI) | P value |
| Non-NAFLD | 1 |  | 1 |  | 1 |  |
| NAFLD | 1.54 (1.23-1.92) | 0.000 | 1.39 (1.09-1.77) | 0.009 | 1.58 (1.21-2.05) | 0.001 |
| **NFS Score** |  |  |  |  |  |  |
| Non-NAFLD | 1 |  | 1 |  | 1 |  |
| NAFLD without advanced fibrosis | 1.33 (0.97-1.80) | 0.074 | 1.20 (0.86-1.65) | 0.283 | 1.37 (0.97-1.94) | 0.074 |
| NAFLD with advanced fibrosis | 1.66 (1.30-2.12) | 0.000 | 1.51 (1.15-1.98) | 0.003 | 1.70 (1.27-2.27) | 0.000 |
| **BARD Score** |  |  |  |  |  |  |
| Non-NAFLD | 1 |  | 1 |  | 1 |  |
| NAFLD without advanced fibrosis | 1.48 (1.08-2.05) | 0.016 | 1.38 (0.99-1.92) | 0.058 | 1.48 (1.04-2.11) | 0.032 |
| NAFLD with advanced fibrosis | 1.56 (1.23-1.98) | 0.000 | 1.39 (1.07-1.82) | 0.016 | 1.63 (1.22-2.17) | 0.001 |
| **FNI Score** |  |  |  |  |  |  |
| Non-NAFLD | 1 |  | 1 |  | 1 |  |
| NAFLD without advanced fibrosis | 3.28 (0.54-19.9) | 0.197 | 2.63 (0.40-17.5) | 0.318 | 3.42 (0.42-27.6) | 0.248 |
| NAFLD with advanced fibrosis | 1.54 (1.23-1.92) | 0.000 | 1.38 (1.08-1.77) | 0.010 | 1.57 (1.21-2.04) | 0.001 |

Model 1 was adjusted for age and sex.

Model 2 was further adjusted for duration of diabetes, antihypertensive drugs, lipid-lowering drugs, antidiabetic agents, smoking, alcohol drinking, body mass index, systolic blood, hemoglobin A1c, and serum levels of triglycerides and low-density lipoprotein based on model 1.

Model 3 was further adjusted for HOMA-IR based on model 2.

HFpEF, heart failure with preserved ejection fraction; NAFLD, nonalcoholic fatty liver disease; OR, odds ratio; CI, confidence interval. HOMA-IR, homeostasis model assessment estimate of insulin resistance; NFS, nonalcoholic fatty liver disease fibrosis score; FNI, fibrotic non-alcoholic steatohepatitis index.

Supplementary table 5. Odds ratios (95% confidence intervals, CI) of the risk of HFpEF from NAFLD and its advanced fibrosis in patients with cardio-cerebrovascular disease

|  | Model 1 |  | Model 2 |  | Model 3 |  |
| --- | --- | --- | --- | --- | --- | --- |
|  | OR (95% CI) | P value | OR (95% CI) | P value | OR (95% CI) | P value |
| Non-NAFLD | 1 |  | 1 |  | 1 |  |
| NAFLD | 0.80 (0.45-1.42) | 0.450 | 0.99 (0.52-1.90) | 0.974 | 1.05 (0.52-2.15) | 0.891 |
| **NFS Score** |  |  |  |  |  |  |
| Non-NAFLD | 1 |  | 1 |  | 1 |  |
| NAFLD without advanced fibrosis | 0.76 (0.20-2.91) | 0.690 | 0.97 (0.23-4.12) | 0.964 | 1.64 (0.37-7.36) | 0.516 |
| NAFLD with advanced fibrosis | 0.81 (0.45-1.46) | 0.483 | 0.99 (0.51-1.95) | 0.982 | 0.98 (0.46-2.07) | 0.953 |
| **BARD Score** |  |  |  |  |  |  |
| Non-NAFLD | 1 |  | 1 |  | 1 |  |
| NAFLD without advanced fibrosis | 1.08 (0.40-2.86) | 0.879 | 1.34 (0.46-3.86) | 0.591 | 1.08 (0.32-3.67) | 0.899 |
| NAFLD with advanced fibrosis | 0.74 (0.39-1.38) | 0.337 | 0.90 (0.44-1.83) | 0.767 | 1.04 (0.48-2.27) | 0.919 |
| **FNI Score** |  |  |  |  |  |  |
| Non-NAFLD | 1 |  | 1 |  | 1 |  |
| NAFLD without advanced fibrosis | - | - | - | - | - | - |
| NAFLD with advanced fibrosis | 0.86 (0.49-1.53) | 0.609 | 0.99 (0.52-1.90) | 0.974 | 1.05 (0.52-2.15) | 0.891 |

Model 1 was adjusted for age and sex.

Model 2 was further adjusted for duration of diabetes, antihypertensive drugs, lipid-lowering drugs, antidiabetic agents, smoking, alcohol drinking, body mass index, systolic blood, hemoglobin A1c, and serum levels of triglycerides and low-density lipoprotein based on model 1.

Model 3 was further adjusted for HOMA-IR based on model 2.

HFpEF, heart failure with preserved ejection fraction; NAFLD, nonalcoholic fatty liver disease; OR, odds ratio; CI, confidence interval. HOMA-IR, homeostasis model assessment estimate of insulin resistance; NFS, nonalcoholic fatty liver disease fibrosis score; FNI, fibrotic non-alcoholic steatohepatitis index.

Supplementary table 6. Odds ratios (95% confidence intervals) of the risk of HFpEF from NAFLD and its advanced fibrosis after excluding smokers.

|  | Model 1 |  | Model 2 |  | Model 3 |  |
| --- | --- | --- | --- | --- | --- | --- |
|  | OR (95% CI) | P value | OR (95% CI) | P value | OR (95% CI) | P value |
| Non-NAFLD | 1 |  | 1 |  | 1 |  |
| NAFLD | 1.32 (1.40-1.68) | 0.022 | 1.20 (0.92-1.56) | 0.191 | 1.39 (1.04-1.85) | 0.025 |
| **NFS Score** |  |  |  |  |  |  |
| Non-NAFLD | 1 |  | 1 |  | 1 |  |
| NAFLD without advanced fibrosis | 1.33 (0.95-1.87) | 0.098 | 1.21 (0.84-1.74) | 0.307 | 1.47 (0.99-2.17) | 0.055 |
| NAFLD with advanced fibrosis | 1.32 (1.00-1.72) | 0.046 | 1.19 (0.88-1.60) | 0.254 | 1.35 (1.0-1.86) | 0.063 |
| **BARD Score** |  |  |  |  |  |  |
| Non-NAFLD | 1 |  | 1 |  | 1 |  |
| NAFLD without advanced fibrosis | 1.16 (0.80-1.70) | 0.430 | 1.06 (0.72-1.57) | 0.775 | 1.12 (0.73-1.71) | 0.597 |
| NAFLD with advanced fibrosis | 1.38 (1.07-1.78) | 0.014 | 1.25 (0.94-1.67) | 0.129 | 1.51 (1.11-2.07) | 0.009 |
| **FNI** |  |  |  |  |  |  |
| Non-NAFLD | 1 |  | 1 |  | 1 |  |
| NAFLD without advanced fibrosis | 1.65 (0.17-16.1) | 0.667 | 1.15 (0.09-14.8) | 0.914 | 1.34 (0.09-20.1) | 0.835 |
| NAFLD with advanced fibrosis | 1.35 (1.06-1.71) | 0.015 | 1.20 (0.92-1.56) | 0.191 | 1.39 (1.04-1.86) | 0.025 |

Model 1 was adjusted for age and sex.

Model 2 was further adjusted for duration of diabetes, antihypertensive drugs, lipid-lowering drugs, antidiabetic agents, alcohol drinking, body mass index, systolic blood, hemoglobin A1c, and serum levels of triglycerides and low-density lipoprotein based on model 1.

Model 3 was further adjusted for HOMA-IR based on model 2.

HFpEF, heart failure with preserved ejection fraction; NAFLD, nonalcoholic fatty liver disease; OR, odds ratio; CI, confidence interval. HOMA-IR, homeostasis model assessment estimate of insulin resistance.

Supplementary table 7. Odds ratios (95% confidence intervals) of the independent and joint associations of NAFLD status and its advanced stage based on BARD score and diabetic care goal attainments for HFpEF risk.

|  | Non-NAFLD | NAFLD without advanced fibrosis | NAFLD with advanced fibrosis | *P* interaction |
| --- | --- | --- | --- | --- |
| **HbA1c** |  |  |  | 0.0302 |
| HbA1c < 7.0% | 1.00 | 1.79 (0.74-4.36) | 2.37 (1.26-4.48) |  |
| HbA1c ≥7.0% | 1.38 (0.91-2.10) | 1.78 (1.08-2.92) | 1.99 (1.28-3.09) |  |
| **BP** |  |  |  | 0.2762 |
| BP < 130/80mmHg | 1.00 | 1.40 (0.76-2.58) | 1.90 (1.24-2.90) |  |
| BP ≥ 130/80mmHg | 1.11 (0.79-1.55) | 1.40 (0.90-2.16) | 1.62 (1.12-2.34) |  |
| **LDL-C** |  |  |  | 0.0211 |
| LDL-C < 100mg/dL | 1.00 | 2.54 (1.14-5.68) | 1.73 (0.94-3.18) |  |
| LDL-C ≥ 100mg/dL | 1.33 (0.85-2.09) | 1.66 (0.99-2.79) | 2.07 (1.30-3.29) |  |
| **LDL-C** |  |  |  | 0.006 |
| LDL-C < 70mg/dL | 1.00 | 1.72 (1.03-2.87) | 1.53 (1.04-2.27) |  |
| LDL-C ≥ 70mg/dL | 1.37 (0.98-1.91) | 1.62 (1.03-2.54) | 2.16 (1.51-3.10) |  |
| **BMI** |  |  |  | 0.3772 |
| BMI < 25 kg/m^2^ | 1.00 | 1.58 (1.01-2.48) | 2.03 (1.42-2.92) |  |
| BMI ≥ 25 kg/m^2^ | 1.16 (0.82-1.64) | 1.31 (0.83-2.07) | 1.53 (1.12-2.09) |  |
| **BMI** |  |  |  | 0.71 |
| BMI < 30 kg/m^2^ | 1.00 | 1.40 (1.00-1.97) | 1.64 (1.26-2.13) |  |
| BMI ≥ 30 kg/m^2^ | 1.43 (0.63-3.24) | - | 1.63 (1.03-2.58) |  |

Models were adjusted for age, sex, duration of diabetes, antihypertensive drug use, lipid-lowering drug use, antidiabetic agent use, smoking, alcohol drinking, BMI, systolic BP, HbA1c, TG, LDL-C and HOMA-IR, except that the variable was used for stratifying subgroups.

NAFLD, nonalcoholic fatty liver disease; HFpEF, heart failure with preserved ejection fraction; HbA1c, hemoglobin A1c; BP, blood pressure; LDL-C, low-density lipoprotein cholesterol; BMI, body mass index; HOMA-IR, homeostasis model assessment estimate of insulin resistance.

Supplementary table 8. Odds ratios (95% confidence intervals) of the independent and joint associations of NAFLD status and its advanced stage based on FNI score and diabetic care goal attainments for HFpEF risk.

|  | Non-NAFLD | NAFLD without advanced fibrosis | NAFLD with advanced fibrosis |
| --- | --- | --- | --- |
| **HbA1c** |  |  |  |
| HbA1c < 7.0% | 1.00 | 11.3 (0.95-135) | 1.89(1.07-3.34) |
| HbA1c ≥ 7.0% | 1.28 (0.86-1.92) | - | 1.78 (1.18-2.68) |
| **BP** |  |  |  |
| BP < 130/80mmHg | 1.00 | - | 1.71 (1.16-2.54) |
| BP ≥ 130/80mmHg | 1.10 (0.79-1.55) | 1.83 (0.15-22.8) | 1.54 (1.09-2.17) |
| **LDL-C** |  |  |  |
| LDL-C < 70mg/dL | 1.00 | - | 1.90 (1.08-3.35) |
| LDL-C ≥ 70mg/dL | 1.35 (0.86-2.11) | 12.7 (1.07-151) | 1.92 (1.22-3.02) |
| **LDL-C** |  |  |  |
| LDL-C< 100mg/dL | 1.00 | - | 1.57 (1.09-2.26) |
| LDL-C≥ 100mg/dL | 1.37 (0.98-1.91) | - | 1.95 (1.39, 2.72) |
| **BMI** |  |  |  |
| BMI < 25 kg/m^2^ | 1.00 | 1.73 (0.14-22.1) | 1.85 (1.35-2.54) |
| BMI ≥ 25 kg/m^2^ | 1.16 (0.82-1.63) | - | 1.47 (1.09-1.97) |
| **BMI** |  |  |  |
| BMI < 30 kg/m^2^ | 1.00 | 1.68 (0.13-21.3) | 1.55 (1.22-1.97) |
| BMI ≥ 30 kg/m^2^ | 1.43 (0.63-3.24) | - | 1.58 (1.0-2.51) |

All analyses were adjusted for age, sex, duration of diabetes, antihypertensive drug use, lipid-lowering drug use, antidiabetic agent use, smoking, alcohol drinking, BMI, systolic BP, HbA1c, TG, LDL-C and HOMA-IR, except that the variable was used for stratifying subgroups.

NAFLD, nonalcoholic fatty liver disease; HFpEF, heart failure with preserved ejection fraction; FNI, fibrotic non-alcoholic

steatohepatitis index; HbA1c, hemoglobin A1c; BP, blood pressure; LDL-C, low-density lipoprotein cholesterol; BMI, body mass index; HOMA-IR, homeostasis model assessment estimate of insulin resistance.
